# Empowerment of frail institutionalized older people for self-care: from administrators’ and staff caregivers’ perspectives

**DOI:** 10.1080/17482631.2021.2022071

**Published:** 2022-01-06

**Authors:** Hsiu-Hui Lin, Ching-Len Yu, Mei-Shu Liou, Hui-Chien Chou, Su-Hsien Chang

**Affiliations:** a Department of Nursing, Kaohsiung Veteran General Hospital Tainan Branch, Tainan, Taiwan ROC; b Department of Environmental Engineering, Kun Shan University, Tainan, Taiwan ROC; c Department of Senior Citizen Services, National Tainan Junior College of Nursing, Tainan, Taiwan ROC

**Keywords:** Older people, empowerment, self-care, long-term care facility, caregivers

## Abstract

**Purpose:**

To investigate the perspectives of administrators and staff caregivers in empowering older people living in long-term care facilities to improve self-care abilities.

**Methods:**

A phenomenology research design was employed to generate data. The purposive sampling method was used to recruit administrator (n = 7) and staff caregiver groups (n =11). Data were collected via face-to-face interviews, observations, and daily recording. The data were then analyzed via content analysis.

**Results:**

The results showed that two elements were of critical importance: professional supports and teamwork. The following professional supports activities were found of positive impacts: allowing residents to perform self-care and improving their mood status. The teamwork was developed via a partnership between staff and family caregivers, and preventing and resolving conflicts in the workplace.

**Conclusion:**

The teamwork could not only reduce the burdens of both staff and family caregivers, but also improve the quality of life and the capacity of older residents. Thus, residents, staffs and family caregivers should work as a team and support older people to perform self-care.

## Introduction

The population is ageing across the world. According to the United Nations World Population Prospects 2019 (2020), persons aged 65 or above accounted for one in 11 (9%) by 2019 and their numbers are projected to increase to one in six (16%) by 2050 (The United Nations Economic and Social Commission for Asia and the Pacific, 2020). Geographically, by 2050, one in four persons living in Europe and Northern America will be aged 65 or over. In East and North Asia, over a third of the population is expected to be 60 years or older, whereas one in four persons will be in that age group in North and Central Asia by 2050. The global trend of population ageing has not only contributed to a rise in the number of people with chronic diseases but also leads to more people living with a disability and needing long-term care services. Older people with comorbidities, polypharmacy, and dependent on personal care services to carry out activities of daily living (ADLs) often lack independence and are more likely to live in an institutional-based long-term care facility, such as nursing home, long-term/nursing/accommodation institutions or assisted-living facility (Barry et al., 2019; Lenaghan, 2019).

Nursing homes provide services to older people with chronic illness and to those who are discharged from hospitals and need continuous skill-nursing care services. The long-term care institutions provide services to older people with chronic illness and to those who need medical and/or rehabilitation services. Nursing institutions and assisted-living facilities provide services to older people who lack abilities to perform activities of daily living, but do not need skill-nursing care services. Accommodation institutions provide services to older people who do not need assistance with activities of daily living and skill-nursing care services. This type of institutions aims to accommodate senior citizens at their own expense, or to shelter senior citizens who have no relatives or their relatives have no abilities to take care of them.

Older people who live in long-term care facilities are frailer and require more assistance in ADLs than elders living in communities. Frailty is a common aspect of vulnerability characterized by deficiencies in physical, cognitive, and/or sensory capacities. Frail older people are at increased risk of physical disabilities, hospitalization, and nursing home admissions (Clegg et al., 2013; Coker et al., 2019). Although frailty often accompanies the ageing process, several factors accelerate its development when older people live in a long-term care facility. These factors include “a place to be idle,” self-perceived incompetence, staff–caregiver-fostered dependency, and family–caregiver-supported dependency (A. Chan et al., 2011; S. H. Chang & Fang, 2004; Coker et al., 2019)

Self-care is one way for older people to gain respect from others and to help themselves to achieve contentment (S. H. Chang et al., 2010). One previous study interviewed 10 nursing home staff and 10 residents to understand their beliefs about self-care. The results showed that staff perceived that performing self-care strengthens older people’s self-esteem, self-confidence, and physical functions. Residents believed that performing self-care brought them happiness and a wish to hasten their return home (S. H. Chang, 2009). Another study used a meta-synthesis to elicit the experiences of self-care among community-dwelling older people. They found that self-care activities were directed towards holistic wellness and the prevention and treatment of ageing. Through self-care activities, older people obtained a sense of satisfaction and self-realization (Lommi et al., 2015). Therefore, self-care activities are settled in a social and relation network that allows older people to take care of themselves and others. Moreover, the other study (Nguyen et al., 2019) examined perceptions of healthy ageing among Korean American, Vietnamese American, and Latino older adults. They found that seven dimensions emerged in participants’ perception of healthy ageing: having good physical health; having good mental health; optimism; acceptance; social connectedness; taking charge of one’s health; and independence and self-worth.

Previous studies already showed self-care is an important dimension for older people to achieve healthy ageing and enjoyment in life. A partnership between older people, staff and family caregivers can benefit older people by facilitating the improvement of self-care performance. Thus far, only limited studies investigated what staff caregivers would do to empower older people to improve self-care abilities and how they would go about it. Therefore, the purpose of this study was to investigate the perspectives of administrators and staff caregivers in the empowerment of older people in long-term care facilities to perform self-care.

## Research questions

1. How does an administrator of long-term care facilities perceive in the empowerment of older people for self-care?
2. How do staff caregivers of long-term care facilities perceive in the empowerment of older people for self-care?
3. How do administrators and staff caregivers conduct the empowerment of older people for self-care daily in long-term care facilities?

## Methodology

This study used a phenomenology research design to generate data on the perspectives of administrators and staff caregivers on the empowerment of older people in long-term care facilities for self-care. The phenomenology design was selected in that it can explore the living experiences of the sample groups and provide a means to uncover the deep understanding of these experiences from the perspective of the groups. To get a better understanding of the perceptions and actions among administrators and staff caregivers, data were collected via face-to-face interviews, observations, and daily recording. Observations and daily recording were conducted during morning care and lunch times to collect subjects’ daily care activities. The data were then analysed via content analysis.

### Sample

The purposive sampling method was used to recruit administrator and staff caregiver groups. The subject of the administrator group had to meet the following selection criteria: 1) the subject is in charge of the organization, the head nurse or the leader of nursing assistants, 2) the subject is in a managerial position of an institutional-based long-term care facility for at least 12 months, and 3) the subject agrees to the interviews being tape-recorded.

The subject of the staff caregiver group had to meet the following selection criteria: 1) the subject is a nursing assistant or a nurse, 2) the subject has worked in a long-term care facility for at least 6 months, and 3) the subject agrees to the interviews being tape-recorded.

For phenomenological studies, Creswell (1998) recommends that the number of subjects should be in the range of 5 to 25. Morse (1994) suggests the number should be at least 6. In this study, 18 subjects were recruited and interviewed.

### Data collection method

This study collected data using an in-depth interviewing technique. The in-depth interviews aimed to elicit the views of the subjects, rather than generalize data to specific subjects or groups. The data were collected through a process of dialogue between the researcher and each subject individually. Then, the researcher collected richly textured details and person-centred narrative data (Kaufman, 1994). The interview guideline is given in Table I, which began with an open-ended question: “What do you perceive the empowerment of older people living in long-term care facilities to improve self-care abilities”?

**Table I.** Guidelines for interviews conducted with administrators and staff caregivers of long-term care facilities

Self-care is defined as “the practice of activities of daily living, including self-bathing, feeding, transferring, dressing, going to the toilet, and maintaining continence.”
**General questions** 1. What do you perceive the empowerment of older people living in long-term care facilities for self-care? 2. What do you do to empower older people in a long-term care facility for self-care? 3. What are facilitators of your empowerment of older people for self-care? 4. What are barriers to your empowerment of older people for self-care?

In this study, all subjects were interviewed in a private room in a long-term care facility where they work, or a location convenient to each subject. The time of the interview was scheduled by the subjects. All interviews were conducted by an investigator in the Chinese language and were audio tape-recorded. Each interview took 30 to 60 minutes. The investigator is female researcher with PhD degree in nursing and was experienced in qualitative research. The interview data were transcribed verbatim before coding.

For ethical considerations, approval from the Human Subjects Committee at the Kaohsiung Veterans General Hospital was obtained before data collection. An explanation of the research purpose and a statement about protecting confidentiality were presented verbally by the investigator to each subject. The interview process posed no known risks. If the subjects felt uncomfortable, the interview would be paused and the subjects could take a rest. The interview would resume when the subject was ready. The subjects were also told that the content of the interview would be kept confidential and their employer would have no right to access it. Besides, every subject was assigned a code number. The code number with subject’s name was destroyed after the data collection process was completed. However, eighteen subjects were recruited and no subject dropped out during the interview process.

### Data analysis

Translations of transcribed interviews were performed after data collection. Mariano (1995) indicated that data collection and data analysis were “hand-in-hand” tasks because initial data analysis could guide later data collection. The reciprocal process continued until no new findings were found, which indicates that the data collection was completed (Jacelon & O’Dell, 2005). In this study, the reciprocal process was adopted.

A three-step data analysis, which was similar to that of Lincoln and Guba (1985), was used in this study. The three steps included: (1) *disaggregating data into the smallest unit*s *of information* that are stand-alone and lack interface with other information, (2) *labelling (coding) these units*, and (3) *progressively sorting them into meaningful categories*. The three-step analysis was performed by the principle investigator (PI) for each interview. After completing the analysis for all interviews, meaningful and accurate categories appeared. In addition, the PI summarized segments of data from interviews into the categories. A *new coding scheme along with* the element*s were also proposed*.

## Results

### Demographic information

Eighteen subjects participated in this study (Administrator group = 7, Staff caregiver group = 11). They were working in intuitional-based long-term care facilities located in southern of Taiwan. The majority of them were female (n = 17). The average age was 40.97 years old (SD = 11.68), and the average working period in the long-term care facility was 99.94 months (Minimum = 11, Maximum = 216). Table II summarizes the characteristics of the study subjects.

**Table II.** Demographic information of study subjects

Variables	Administrators	Staff Caregivers
Age	45.26 (7.20)	38.26 (13.42)
Period of working in the present setting (months)	104.42 (60.06)	51.64 (65.34)
Period of working in the long-term care facility (months)	117.86 (46.01)	88.55 (80.56)
Gender		
Male	0 (0%)	1 (9%)
Female	7 (100%)	10 (91%)
Salary		
20,000–30,000 NT	1 (14.3%)	6 (54.5%)
30,001–40,000 NT	1 (14.3%)	5 (45.5%)
40,000 NT and over	5 (71.4%)	0 (0%)
Years of receiving education		
≦9 years	1 (14.3%)	2 (18.18%)
12–15 years	1 (14.3%)	4 (36.36%)
≧16 years	5 (71.4%)	5 (45.46%)
Marital status		
Single	2 (28.58%)	4 (36.36%)
Married	5 (71.42%)	7 (63.64%)

### Study findings

**Figure 1.**
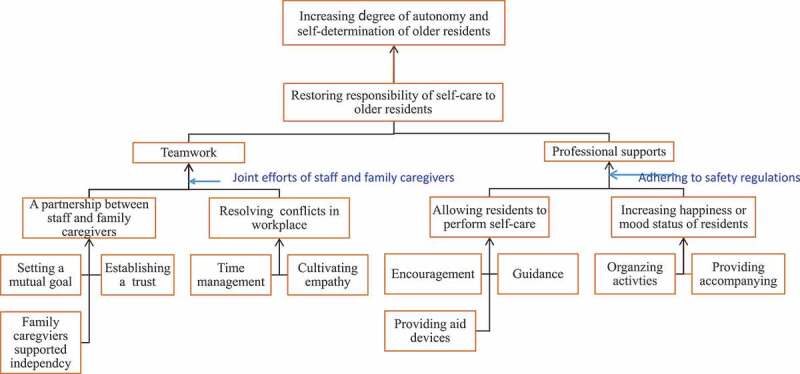
Process of empowering older people living in long-term care facilities for self-care.

As shown in Figure 1, most subjects believed that the goal of the process of the empowerment is to ***return responsibility of self-care to the residents***. Two elements were believed to be of critical importance: *professional support* and *teamwork*. With the two elements, older people in long-term care facilities can *increase autonomy and self- determination*. According to a staff caregiver (#2), “The empowerment of older residents is to allow the elders to make their own decision. A nurse needs to assess self-care abilities of elderly people, such as muscle power, and then provide partial and less assistance in activities of daily performance.” An administrator (#6) indicated that “Each elder has had their own life experiences and lifestyle model. Nursing assistants should not interfere too much (when older people are making a decision). If older persons need assistance, they will inform you”

#### Professional support

During the process of returning the responsibility of self-care to older residents, it is still necessary to provide sufficient professional support to comply with the resident safety regulations. The following activities of professional support were suggested: *allowing residents to perform self-care* and *doing somethings to improve the*ir *happiness or mood status*. When allowing residents to perform ADLs, staff caregivers need to provide *encouragement and guidance*, and *assistive devices*. A staff caregiver (#9) indicated that “ … under (resident) safety regulations … we hope that all residents can maintain their abilities (in performing ADLs). For example, if their right hand is mobile, we will encourage and guide them to dress or undress. Another staff caregiver (#14) stated that “We assess residents’ abilities (in performing self-care). For example, when assisting toileting, we need to assess the resident’s muscle strength of upper limbs and lower limbs, and see if they need an aid device. If they need an aid device (during ADLs), we should provide them with an aid device. In addition, regaining residents’ self-care abilities, giving encouragement and guidance, and providing assistive devices are important during the training phase. All training (to regain abilities in ADLs) should obey the resident safety regulations. and begin with a self-care ability assessment. Otherwise, we (staff caregivers) will get half the result with twice the effort.”

Most administrators and staff caregivers suggested that *organizing activities and providing more company* can increase happiness or improve the mood status of older people. As a result, older people were motivated to learn how to perform self-care. A staff caregiver (#15) indicated that “We took care of many elders (in a long-term care facility) daily. There was little time to accompany them. If someone can talk to or accompany them, their mood will improve. Consequently, their learning motivation (or regaining self-care) will increase.” An administrator (# 5) also indicated that “ … if someone can talk to or accompany them (a resident), and organize activities for them, they will feel happier …. When their mood is improved, this motivates them to learn or regain (performance of ADLs).”

#### Teamwork

Another important element aimed at assisting older residents to return self-care responsibility is *teamwork*. Teamwork was developed via *a partnership between staff and family caregivers*, and *avoiding and resolving conflicts in the workplace*. A partnership between staff and family caregivers is established via *setting a mutual goal, building a trust relationship, and family caregivers supporting the independence of* their relative. An administrator (# 16) indicated that “ … when an older person is placed in a long-term care facility, they need time to adapt to a new environment and life. At that time, we should spend more time with them and demonstrate caring to them and make them feel comfortable and safe. When they trust you, they will become cooperative and follow your guidance and learn (regain or maintain) self-care.

Family caregivers of older residents should trust staff caregivers, and support the independence of their relative. Without the support of family caregivers, it is difficult to assist residents to regain or maintain self-care.” Another administrator (#3) stated that “Family caregivers’ expectations of the self-care improvement affect their attitudes toward supporting the return of self-care responsibility of elders. If the administrator of a long-term care facility and staff caregivers have a mutual goal, and then together they have good communication with family caregivers of residents and empower older people in performing ADLs independently, the older people will be able to regain or maintain ADLs performance.” Similarly, an administrator (#5) stated that “We share ideas and opinions during the lunch break, and to agree a mutual goal on how to empower elders to perform self-care. We also advocate this idea to family caregivers when they visit their relative.”

Teamwork frequently requires staff caregivers to resolve conflicts in the workplace. To resolve conflicts, staff caregivers need to learn *time management* and *cultivating empathy*. A staff caregiver indicated that “ … Based on the workflow of long-term care facilities, staff caregivers have a poor grasp of time, which leads to the fact that if the residents are not authorized to perform self-care, they (staff caregivers) will be unable to complete their work as scheduled. This problem can be solved if staff caregivers empower the resident for self care. For example, staff caregivers can work as a team to assist a heavier resident, to get off the bed, at the same time, to have another elder brush their own teeth. Good time management can be obtained via empowering residents in self-care performance.” *Another* staff caregiver (# 9) stated that “If a staff caregiver frequently puts herself/himself in someone else’s shoes, gradually they cultivate empathy. They will assist the residents with walking and balance if they have time. Day by day, perhaps, older people will not only be able to walk again, but also improve their self-confidence.”

## Discussion

The present results indicated that when older residents received professional support from a team of staff and family caregivers to allow them perform self-care, they increased the degree of their autonomy and self-determination. These results were consistent with those of two previous studies (S. H. Chang, 2009; Halvorsen et al., 2020). It should be stressed that the empowerment of an individual is a cognitive motivation generated from within, as indicated by Quinn and Spreitzer (1997). Also, VanderPlaat (1999) stated that empowerment is not something given, but something taken in equal relations. In other words, empowerment can be viewed as a helping process of making an individual act differently, not just the distribution of power (Halvorsen et al., 2020). Therefore, support for older residents to perform self-care by nursing home staff caregivers and family members of residents should be encouraged. Autonomy and self-determination of an older resident is a symbol of empowerment, which reflects the caring quality of this professional team. With continuous efforts of the team, older residents can develop self-esteem, life satisfaction, self-confidence, and maintain their physical functions and happiness (S. H. Chang, 2009).

Chan et al. (2020) conducted a study to understand older patients’ perception of engagement in functional self-care during hospitalization. They found that facilitators for older patients to perform self-care should include patients’ positive mindset and an age-friendly environment. Barriers for older patients to perform self-care were healthcare-imposed restrictions and nurses’ fear of patients falling. Hence, the notion of “good” patient can be either facilitator or barrier in their self-care engagement.

This study also found that teamwork between staff and family caregivers can not only resolve workplace conflict among staff caregivers but also build a partnership aimed at empowering older people for self-care. These results were similar to several previous studies. For example, Halskov et al. (2016) examined the perspectives on the role and responsibilities in the collaborations with home care nurses among 14 older patients with chronic illness. They found that some patients played a host role that would determine the conditions for the home care nurse’s visits and expect the nurses to act as a guest. The guest role of the nurses showed courtesy and good manners and respected the house rules, daily routine and preferences of the patient. This was how the home care nurse empowered older patients with clinical illness and was only partly involved in care and treatment.

Wang et al. (2018) also indicated that staff awareness and capacity to empower residents via care plans would improve their quality of life. Similarly, Coker et al. (2019) interviewed 22 staff caregivers in the community to reveal their perceptions of frailty. They reported that frailty was associated with increasing age, especially the vulnerability of older adults. To assess and manage frailty, a holistic approach required an interdisciplinary team of specialists. However, results of the study indicated that empowerment of older people living in long-term care facilities should be supported if safety concerns are not an issue. In safe conditions and environment, staff and family caregivers empowered older residents to perform self-care to increase their happiness.

## Conclusion

To empower elders living in long-term care facilities, older residents, staff and family caregivers should work together and support elders to perform self-care. The teamwork through the joint efforts of staff caregiver, family caregivers, and older resident efforts can strengthen self-care abilities of older people. As a results, not only were the burdens of both staff and family caregivers reduced, but also the quality of life and the capacity of older residents were improved. This is obviously of great benefit to achieve conformity with nature, which is the way to enhance the health and wellness of older residents. For further investigation, we are currently developing interventions on the self-care performance of older residents empowered by staff and family caregivers.
